# Identification of genes associated with fatty acid biosynthesis based on 214 safflower core germplasm

**DOI:** 10.1186/s12864-023-09874-5

**Published:** 2023-12-11

**Authors:** Kangjun Fan, Yonghua Qin, Xueli Hu, Jindong Xu, Qingzhi Ye, Chengyang Zhang, Yangyang Ding, Gang Li, Yan Chen, Jiao Liu, Peiqi Wang, Zunhong Hu, Xingchu Yan, Hairong Xiong, Hong Liu, Rui Qin

**Affiliations:** 1 Hubei Provincial Key Laboratory for Protection and Application of Special Plant Germplasm in Wuling Area of China, College of Life Sciences, South-Central MinZu University, Wuhan, 430074 China; 2 Industrial Crop Research Institute of Yunnan Academy of Agricultural Sciences, Kunming, 650205 China; 3 Oil Crops Research Institute, Chinese Academy of Agricultural Sciences (CAAS), Wuhan, China

**Keywords:** *Carthamus tinctorius* L., Genetic Diversity, Fatty acid composition, Transcriptome sequencing, Fatty acid biosynthesis

## Abstract

**Background:**

Safflower (*Carthamus tinctorius* L.) is an oilseed crop with substantial medicinal and economic value. However, the methods for constructing safflower core germplasm resources are limited, and the molecular mechanisms of lipid biosynthesis in safflower seeds are not well understood.

**Results:**

In this study, 11 oil-related quantitative traits and 50 pairs of InDel markers were used to assess the diversity of a collection of 605 safflower germplasms. The original safflower germplasm exhibited rich phenotypic diversity, with high variation for most of the phenotypic traits under investigation. Similarly, high genetic diversity was evaluated in the original germplasm, in which the mean Shannon’s information index (*I*), observed heterozygosity (*H*_*0*_), and expected heterozygosity (*He*) were 0.553, 0.182, and 0.374, respectively. Four subgroups with strong genetic structures were identified and a core germplasm of 214 cultivars was constructed, which is well represented in the original germplasm. Meanwhile, differential expression analysis of the transcriptomes of high and low linoleic acid safflower varieties at two stages of seed development identified a total of 47 genes associated with lipid biosynthesis. High expression of the genes KAS II and *SAD* enhanced the synthesis and accumulation of oleic acid, while FAD genes like *FAD2* (Chr8G0104100), *FAD3*, *FAD7* and *FAD8* promoted the consumption of oleic acid conversion. The coordinated regulation of these multiple genes ensures the high accumulation of oleic acid in safflower seed oil.

**Conclusions:**

Based on these findings, a core germplasm of 214 cultivars was constructed and 47 candidate genes related to unsaturated fatty acid biosynthesis and lipid accumulation were identified. These results not only provide guidance for further studies to elucidate the molecular basis of oil lipid accumulation in safflower seeds, but also contribute to safflower cultivar improvements.

**Supplementary Information:**

The online version contains supplementary material available at 10.1186/s12864-023-09874-5.

## Background

Safflower (*Carthamus tinctorius* L., 2*n* = 24) is an annual self-fertilizing crop of the Asteraceae family, which originated from the Fertile Crescent approximately 4,000 years ago [1]. Ordinary safflower seed kernel after dehulling contains 30%–35% oil, mainly in the form of triacylglycerol (TAG). Safflower seed oil is typically composed of 76%–83% linoleic acid (C18:2), 9%–12% oleic acid (C18:1), 5.5%–6.4% palmitic acid (C16:0), 1.2%–3.1% stearic acid (C18:0), and several other minor fatty acids [2]. Among these compounds, linoleic acid and oleic acid are the two main unsaturated fatty acids that can lower blood cholesterol level [3]. In addition, hydroxysafflor yellow A (HSYA) is a flavonoid compound that is only present in safflower petals and is widely used in pharmaceuticals for the treatment of cardiovascular diseases and a natural dye in food and cosmetics industries [4, 5]. However, the most cultivated safflower cultivars still have certain problems such as low safflower seed yield and susceptibility to severe effects of multiple biotic stresses, thus greatly limiting safflower seed oil production.

The conservation of germplasm utilization has been improved by establishing the core germplasm to capture the maximum variability prevalent throughout the germplasm and thus promote the efficient use of germplasm resources [6]. Currently, molecular markers such as Amplified Fragment Length Polymorphism (AFLP) and Simple Sequence Repeats (SSR) are commonly used to study the development of core safflower germplasm [7, 8]. However, most of these studies are limited to collections in certain regions with small sample sizes, resulting in a lack of global representation and comprehensive genomic information. In contrast, Insertion/deletion (InDel) and Single Nucleotide Polymorphisms (SNPs) become the most prevalent markers due to their efficiency, low cost and simplicity of detection [9]. Therefore, construction of a representative and accurate core germplasm dataset can be achieved by combining InDel markers with phenotypic data, thereby providing a rich resource for future research on the biosynthesis pathway of fatty acids.

The investigations of the molecular mechanisms involved in lipid metabolism are critical for the genetic engineering of safflower to increase its oil accumulation level or change the oil composition. Plant lipid biosynthesis is a complex network of regulated metabolism that involves three main stages, namely, ab initio synthesis of lipids, acyl modifications, and TAG biosynthesis [10]. The molecular mechanism of oil biosynthesis has been revealed in many plants such as soybean, peanut, sesame, and rapeseed, and many key genes associated with oil biosynthesis have been identified to elucidate the genetic basis of seed development and oil accumulation [11–14]. The research on lipid synthesis in safflower is quite limited. Chen et al. reported that two lipase genes (HH-026818-RA and HH-025320) may participated in glycerolipid metabolism and fatty acid degradation and lead to the degradation of oil bodies (TAG) and membrane lipids that integrate proteome and lipidome analysis[15]. Li et al. found significant expression changes in the *SAD* and *FAD2-1* genes at 14–18 DAF at the expression level by using de novo assembled transcriptome analysis, and no additional data were available to determine the contribution of the genes to seed oil biosynthesis and fatty acid accumulation[16]. The nuclear factor YB of *Carthamus tinctorius* L. increased the content of unsaturated fatty acids by regulating the expression of genes involved in fatty acid synthesis and oil accumulation [17]. However, the molecular mechanisms involved in lipid metabolism in safflower remain unknown.

The chromosome-scale reference genome of safflower has been reported, and the fatty acid desaturase 2 (*FAD2*) in high linoleic acid safflower were enriched for those predicted to be involved in lipid metabolism and transport based on comparative genomic analyses [18]. In the present study, the genetic diversity of 605 safflower germplasm was assessed using InDel markers and oil phenotype data construct a core germplasm as a foundation for safflower breeding. Secondly, transcriptome data pathways and key genes associated with oil accumulation during safflower seed developmental stages were investigated. The results will improve the understanding of the regulatory mechanisms of oil biosynthesis in safflower seed and will provide a useful genetic resource toward the production of high-quality and high-yield safflower seed oils.

## Result

### Fatty acid compositions of safflower seeds

**Fig. 1:**
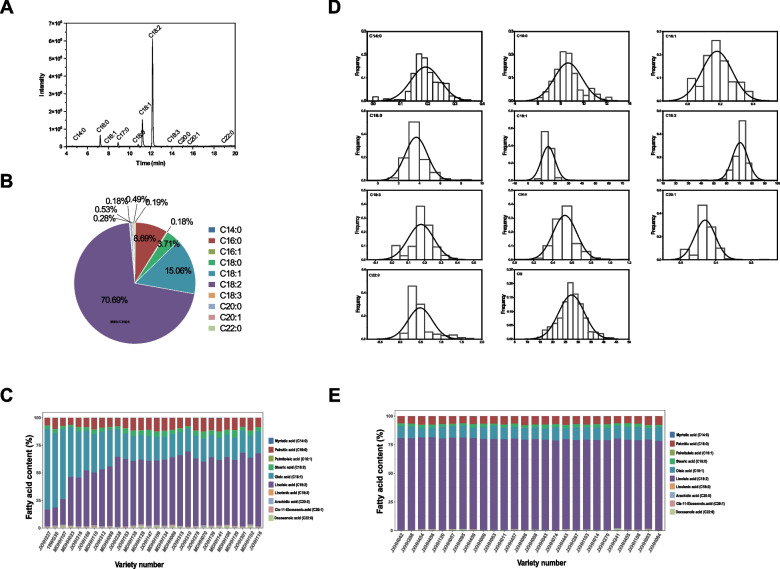
Determination of fatty acid composition of safflower. **A** Representative diagram of GC–MS analysis. **B** Fatty acid composition in 605 safflower germplasm. **C** Varieties with > 20% oleic acid content. **D** Normal distribution of 11 quantitative traits. **E** Varieties with > 78% linoleic acid content. Lipid substrates are abbreviated as follows: C14:0, myristic acid; C16:0, palmitic acid; C16:1, palmitoleic acid; C18:0, stearic acid; C18:1, oleic acid; C18:2, linoleic acid; C18:3, linolenic acid; C20:0, arachidic acid; C20:1, cis-11-eicosenoic acid; C22:0, docosanoic acid

The fatty acid composition of 605 safflower germplasm resources collected around the world was investigated by gas chromatograph-mass spectrometry (GC–MS) (Fig. 1A). Ten kinds of fatty acids were detected, of which unsaturated fatty acids accounted for more than 90% of the oil concentration (Fig. 1B). Linoleic acid was the highest unsaturated fatty acid in safflower seed oil, followed by oleic acid. Among the 605 safflower materials, 28 had 20% or more oleic acid and 26 had 78% or more linoleic acid (Fig. 1C, 1E). The frequency distribution of the composition of 10 fatty acid components and oil content showed that the phenotypic data were close to the normal distribution model, as illustrated in Fig. 1D. The analysis of fatty acid composition and oil content of safflower showed that the differences in oleic acid and linoleic acid contents were large, while the differences in palmitic acid and stearic acid in terms of total fatty acid content among different varieties were not remarkable (Additional file 1: Table S1). The variation coefficients of fatty acids among the 605 safflower materials ranged from 8.64% to 60.10%, in which the largest coefficient of variation was observed for behenic acid, the smallest coefficient of variation was observed for linoleic acid, and 18.33% coefficient of variation was observed for oil content. The genetic diversity indices of 11 quantitative traits ranged from 1.50 to 2.00, indicating that different safflower varieties had different degrees of differences in terms of fatty acid composition and exhibited rich genetic variability.

**Fig. 2:**
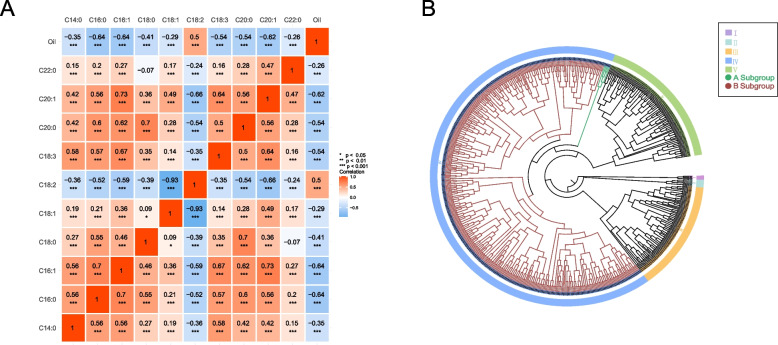
Fatty acid composition analysis of 605 safflower germplasm. **A** Quantitative trait correlation analysis. **B** Cluster analysis based on oleic and linoleic acid content

Different degrees of correlations were observed among the 11 traits, and most of them showed significant or highly significant correlations with each other (Fig. 2A). The content of oleic acid showed the highest negative correlation with linoleic acid (*r* =  − 0.93 ***). The content of palmitoleic acid had a highly positive correlation with eicosenoic acid (*r* = 0.73 ***). A strong correlation was observed between palmitic acid (C16:0) and stearic acid (C18:0, *r* = 0.55 ***) possibly because C16:0 is a substrate for C18:0 synthesis.

The results of the principal component analysis of fatty acid composition and oil content of 605 safflower germplasm reflected the linear combination relationship between them. The first three principal components contained the main information about fatty acid composition with a cumulative contribution of 74.42%. Palmitoleic acid, linolenic acid, and eicosenoic acid had high positive loadings in the first principal component, while linoleic acid had high negative loadings, and these compounds are all unsaturated fatty acids. In the second principal component, linoleic acid had high positive loadings, while oleic acid had high negative loadings. In the third principal component, docosanoic acid had high positive loadings, while stearic acid had high negative loadings. In addition, oleic acid had the highest negative loading (− 0.647), and linoleic acid had the highest positive loading (0.447) among these unsaturated fatty acid components. Therefore, the two fatty acid components of oleic acid and linoleic acid can accurately reflect the fatty acid components of different varieties of safflower seed oil (Additional file 1: Table S2 ).

The dendrogram based on UPGMA analysis by using both oleic and linoleic acid components was used to divide the collection into five groups. Cluster I contains three germplasm, and it is mainly characterized by oleic acid content of 63% or more; Cluster II contains six germplasm, and it is mainly characterized by oleic acid content of 33%–45%; Cluster III contains 84 germplasm, and it is mainly characterized by linoleic acid content of 58%–69%; Cluster IV contains 409 germplasm, and it is mainly characterized by linoleic acid content in the range of 70–74%. Cluster IV can be further divided into two subgroups based oleic acid only (A, B): subgroup A contains 5 germplasm characterized by oleic acid content of 13–19%, and subgroup B contains 404 germplasm characterized by oleic acid content of 9–13%. However, we could not observe any profound variations linoleic acid content within Cluster IV. Cluster V contains 103 germplasm, and it is mainly characterized by linoleic acid content of 75% or more (Fig. 2B).

The genetic diversity and genetic structure of 605 safflower germplasm were analyzed based on 50 pairs of InDel molecular markers, and the results showed that 100 alleles were detected in 605 safflower germplasm (Table 1), and the average observed number of alleles was 2. The mean Shannon’s information index (*I*), observed heterozygosity (*H*_*0*_), and expected heterozygosity *(H*_*e*_*)* were 0.553, 0.182, and 0.374, respectively. The polymorphism content (PIC) ranged from 0.151 (Loci9) to 0.375 (Loci10, Loci19, Loci14) with a mean of 0.311, which were reasonably informative based on the classification of Botstein et al. [19]. These results indicated a relatively reasonable level of genetic diversity in 605 safflower germplasm.

**Table 1:** Genetic diversity analysis of 50 pairs of InDel primers

Locus	*N*_*A*_	*N*_*E*_	*I*	*H*_*0*_	*H*_*E*_	*PIC*
Loci1	2	1.569	0.549	0.245	0.363	0.314
Loci2	2	1.174	0.280	0.106	0.148	0.164
Loci3	2	1.644	0.580	0.217	0.392	0.319
Loci4	2	1.933	0.676	0.256	0.483	0.368
Loci5	2	1.368	0.440	0.133	0.269	0.259
Loci6	2	1.544	0.537	0.147	0.352	0.281
Loci7	2	1.735	0.615	0.200	0.424	0.353
Loci8	2	1.799	0.636	0.198	0.444	0.354
Loci9	2	1.130	0.230	0.046	0.115	0.151
Loci10	2	1.998	0.693	0.135	0.499	0.375
Loci11	2	1.486	0.508	0.147	0.327	0.292
Loci12	2	1.273	0.371	0.065	0.214	0.249
Loci13	2	1.776	0.629	0.203	0.437	0.351
Loci14	2	1.992	0.691	0.166	0.498	0.375
Loci15	2	1.445	0.487	0.104	0.308	0.298
Loci16	2	1.955	0.682	0.128	0.489	0.370
Loci17	2	1.670	0.591	0.151	0.401	0.339
Loci18	2	1.344	0.424	0.124	0.256	0.247
Loci19	2	1.999	0.693	0.217	0.500	0.375
Loci20	2	1.472	0.501	0.179	0.321	0.301
Loci21	2	1.987	0.690	0.250	0.497	0.373
Loci22	2	1.813	0.641	0.190	0.449	0.351
Loci23	2	1.148	0.252	0.097	0.129	0.176
Loci24	2	1.160	0.265	0.073	0.138	0.191
Loci25	2	1.919	0.672	0.282	0.479	0.372
Loci26	2	1.891	0.664	0.274	0.471	0.354
Loci27	2	1.683	0.596	0.225	0.406	0.332
Loci28	2	1.925	0.674	0.269	0.481	0.368
Loci29	2	1.819	0.642	0.234	0.450	0.355
Loci30	2	1.267	0.367	0.243	0.211	0.194
Loci31	2	1.355	0.431	0.113	0.262	0.282
Loci32	2	1.200	0.306	0.097	0.167	0.205
Loci33	2	1.203	0.309	0.000	0.169	0.166
Loci34	2	1.325	0.410	0.076	0.245	0.247
Loci35	2	1.342	0.423	0.134	0.255	0.249
Loci36	2	1.827	0.645	0.262	0.453	0.352
Loci37	2	1.806	0.638	0.235	0.446	0.338
Loci38	2	1.952	0.681	0.235	0.488	0.374
Loci39	2	1.902	0.667	0.184	0.474	0.354
Loci40	2	1.990	0.691	0.188	0.498	0.374
Loci41	2	1.735	0.615	0.174	0.424	0.346
Loci42	2	1.867	0.657	0.153	0.464	0.348
Loci43	2	1.426	0.476	0.266	0.299	0.280
Loci44	2	1.956	0.682	0.248	0.489	0.369
Loci45	2	1.945	0.679	0.211	0.486	0.373
Loci46	2	2.000	0.693	0.315	0.500	0.372
Loci47	2	1.436	0.482	0.170	0.304	0.285
Loci48	2	1.501	0.516	0.349	0.334	0.307
Loci49	2	2.000	0.693	0.206	0.500	0.374
Loci50	2	1.907	0.669	0.206	0.476	0.368
Mean	2	1.652	0.553	0.182	0.374	0.311

Note: *N*_*A*_ number of observed alleles; *N*_*E*_ number of valid alleles

**Fig. 3:**
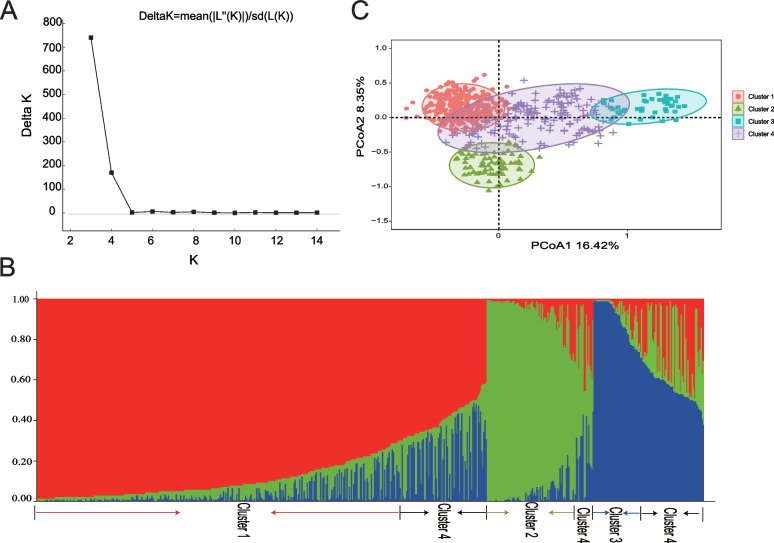
Analysis of population structure of 605 safflower germplasm. **A** Delta K that shows the population size, **B** Population genetic structure based on the Bayesian clustering model with four subgroups at *K* = 3. C: Score plot generated using PCoA

Population structure, genetic diversity, and correlation analysis of safflower germplasm were carried out. All these polymorphic InDel loci (50 pairs) were used to estimate the genetic diversity of 605 safflower germplasm. The number of subpopulations of safflower germplasm for testing was identified using Structure v2.3.4 software. At *K* = 3, the maximum Δ*K* value was recorded (Fig. 3A), indicating the presence of three subpopulations in the test safflower germplasm. With the membership probabilities of ≥ 0.70, 328 accessions (54.21%) were assigned to Cluster 1,76 accessions (12.56%) were assigned to Cluster 2, 44 accessions (7.27%) were assigned to Cluster 3 and 157 accessions (25.95%) were assigned to retained in the admixed group (Cluster 4) (Fig. 3B).

Analysis of molecular variance (AMOVA) was carried out, and the genetic differentiation coefficient (*F*_*ST*_) was calculated to investigate population differentiation among inferred subpopulations. AMOVA results revealed that at the species level, inter-safflower subgroup variation accounted for 22% of the total variation, with most of the variation present within subgroups (*F*_*st*_ = 0.218, *P* value = 0.001, Additional file 1: Table S4), and inter-subgroup differentiation reached a significant level. *F*_*st*_ ranged from 0.069 to 0.425, and showed great genetic differentiation between Cluster 1 and Cluster 3, and between Cluster 2 and Cluster 3 (Additional file 1: Table S5). Furthermore, based on the principal coordinate analysis (PCoA) of 605 safflower germplasm on genetic distance, four clusters could be clearly distinguished (Fig. 3C).

### Construction of core germplasm

**Fig. 4:**
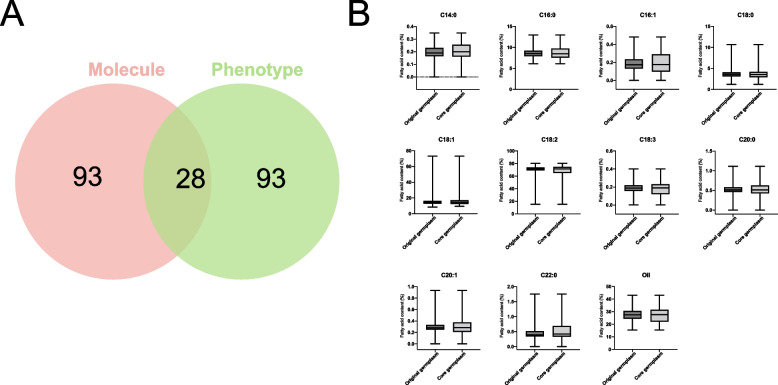
Statistical analysis of original and core germplasm. **A** Venn diagram of two types of data for constructing core germplasm. **B** Box line plot of mean, standard deviation, maximum, and minimum values of 11 quantitative traits

The core germplasm constructed based on InDel markers (Core 1) and the core germplasm constructed for 11 quantitative traits (Core 2) had a 50% replication rate. By combining core 1 and core 2 as the final core germplasm, 214 core germplasm of safflower cultivars were obtained (Fig. 4A, Additional file 1: Table S6). The evaluation using InDel markers demonstrated that the 214 safflower core germplasm are more representative than that of the original population (Additional file 1: Table S7). The original and core germplasm for 11 quantitative traits were compared via statistical analysis (Fig. 4B, Additional file 1: Table S8). Based on the results, the mean difference percentage (MD = 18.18%), periodic rate of range (CR = 132.79%), changeable rate of the coefficient of variation (VR = 99.84%), and variance difference percentage (VD = 100%) of the traits were estimated, and the results met the evaluation criteria proposed by Hu et al. [20]. Thus, the core germplasm had good diversity.

Association analysis was performed using GLM model for 11 oil traits in the 605 safflower germplasm. The results of GLM analysis showed that 32 InDel loci were associated with safflower fatty acids (p < 0.01), in which phenotypic variance explained between 1.55% and 4.26% of the variation. Loci 33 locus had the highest phenotypic explanation for 11-eicosenoic acid (4.26%), while myristic acid, palmitic acid, palmitoleic acid, stearic acid, linoleic acid, 11-eicosenoic acid, and oil contents were detected at the loci 23 locus, and oleic acid and linoleic acid were detected at the loci 30 locus (Additional file 1: Table S9).

### Dynamic changes in fatty acid content and associated genes in during safflower seed development

The composition of fatty acids in the two stages of seed development in low linoleic (LL) cultivar “AnHui” and high linoleic (HL) cultivar “France” safflower cultivar were determined by GC–MS. Eight fatty acids were detected, and the linoleic acid content of HL safflower was higher than that of LL safflower during the two periods of safflower seed development (Additional file 2: Fig. S1).

**Fig. 5:**
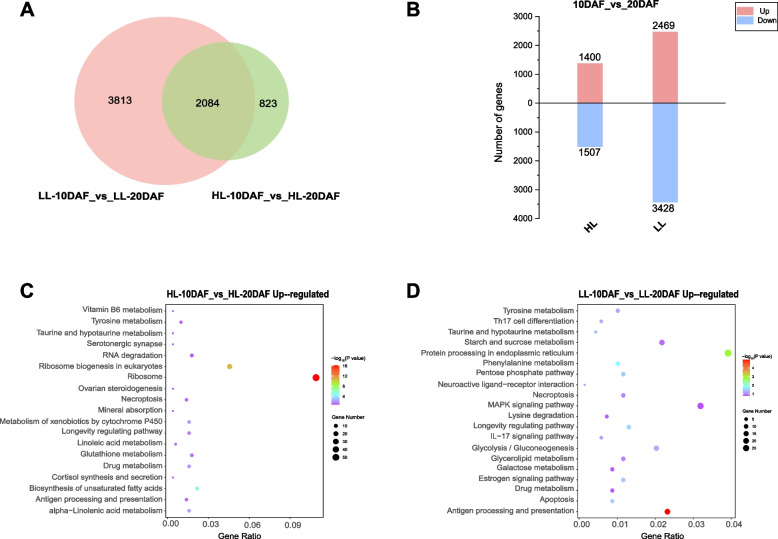
Analysis of differentially expressed genes (DEGs) in two safflower varieties at different seed development stages. **A** Venn diagram that indicates the number of differential genes. **B** Number of up- and downregulated differential genes. **C** Enriched DEGs of Kyoto Encyclopedia of Genes and Genomes (KEGG) pathway in HL safflower upregulated genes. **D** Enriched DEGs of KEGG pathway in LL safflower upregulated genes

The dynamics of the transcriptome during safflower seed development was investigated by analyzing the differentially expressed genes (DEGs) of the two varieties at adjacent developmental stages (Fig. 5A, B). The LL safflower had more up- and downregulated DEGs than HL safflower, which was consistent with the fatty acid accumulation pattern, and LL safflower had substantial differences in fatty acid content from 10 to 20 days after flowering (DAF, Additional file 2: Fig. S1). The functional enrichment analysis of DEGs at each stage in LL and HL safflower varieties revealed that the upregulated DEGs at 10 versus 20 DAF were significantly involved in the biosynthesis of unsaturated fatty acids (ko01040), alpha-linolenic acid metabolism (ko00592), and linoleic acid metabolism (ko00591) in HL safflower. In LL safflower, the upregulated DEGs at 10 versus 20 DAF were only involved in glycerolipid metabolism (ko00561) (Fig. 5C, D).

**Fig. 6:**
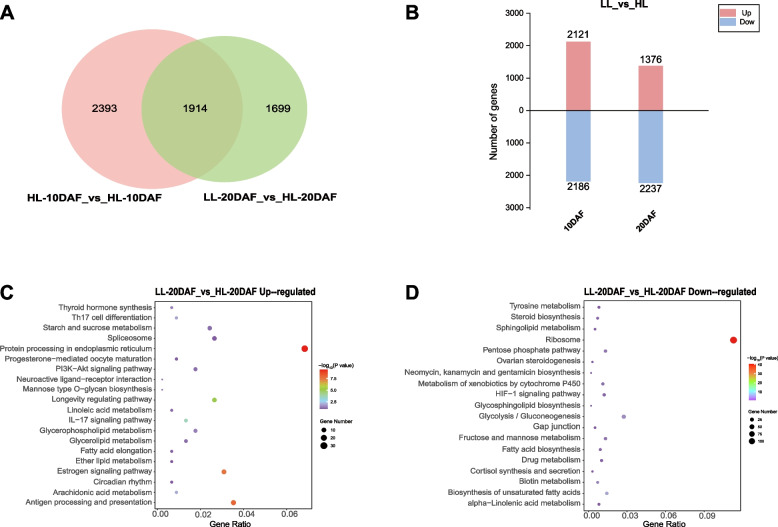
Analysis of differentially expressed genes (DEGs) in the comparison of low linoleic (LL) and high linoleic (HL) safflower. **A** Number of DEGs. **B** Number of up- and downregulated genes in LL relative to the corresponding expression levels in HL at each seed developmental stage. **C** Enriched KEGG pathway classification of LL and HL upregulated DEGs in 20DAF. **D** Enriched KEGG pathway classification of LL and HL downregulated DEGs in 20DAF

The key genes involved in the high- and low-linoleic varieties were determined by analyzing the DEGs at the same developmental stages (Fig. 6A, B). A total of 4,092 DEGs were identified with significant differences in the comparison between LL and HL after removing duplicate DEGs. A subsequent KEGG enrichment analysis was performed to determine the gene expression profile of LL versus HL developmental stages, and the lipid metabolism pathway was focused on (Fig. 6C, D). The upregulated DEGs are involved in arachidonic acid metabolism (ko00590), glycerophospholipid metabolism (ko00564), glycerolipid metabolism (ko00561), linoleic acid metabolism (ko00591), and fatty acid extension (ko00062), while the downregulated DEGs are involved in biosynthesis of unsaturated fatty acids (ko01040), sphingolipid metabolism (ko00600), glycolipid biosynthesis (ko00603), and α-linolenic acid metabolism (ko00592). These results indicate that the linoleic acid content of LL varieties at 10 DAF and 20 DAF was lower than that of HL varieties (Additional file 2: Fig. S1).

**Fig. 7:**
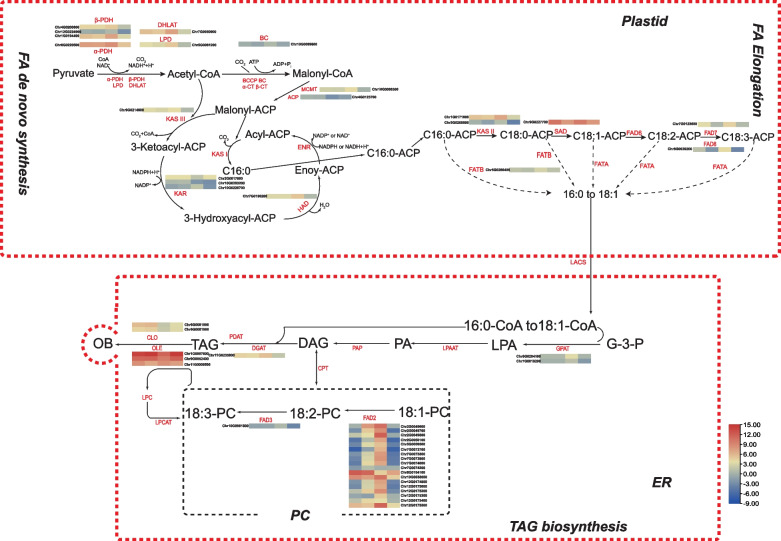
Pathways of differentially expressed genes (DEGs) involved in fatty acid and triacylglycerol (TAG) biosynthesis. These differential genes are placed in the pathway, in which the first two columns represent HL safflower at 10DAF and 20DAF, and the last two columns represent LL safflower at 10DAF and 20DAF; the rows indicate different lipid-associated DEGs, which were placed near the enzyme names to indicate differences in the expression of genes that encode the enzymes at different stages. The enzyme/protein abbreviations are as follows: This model was modified from the model published by [51]

**Fig. 8:**
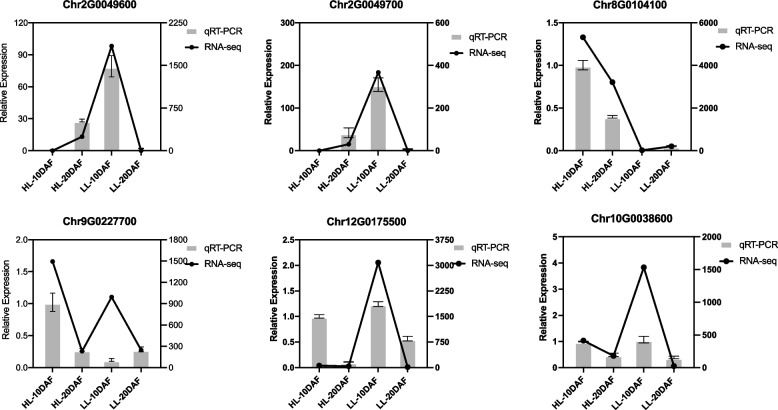
qRT-PCR validation of the expression levels of differentially expressed genes related to lipid synthesis pathway in low linoleic and high linoleic safflower varieties. Values are expressed as means ± SEM (*n* = 3)

Differential expression analysis of lipid synthesis-related genes at different stages of LL and HL safflower varieties identified 47 genes related to lipid biosynthesis (Fig. 7, Table S9). In fatty acid biosynthesis, 14 DEGs were identified, of which six DEGs encoded pyruvate dehydrogenase (PDH), 1 DEG encoded acetyl-CoA carboxylase (ACCase), 1 DEG encoded malonyl-CoA ACP transacylase (MCAT*)*, 3 DEGs encoded 3-oxoacyl-ACP reductase (KAR), and 4 DEGs encoded other components of FA synthase. Fragments per kilobase per million (FPKM) values indicated that these genes involved in the ab initio synthesis of fatty acids had at least one significantly upregulated isozyme during the phase of rapid lipid accumulation, and this result is consistent with the ongoing lipid biosynthesis at this stage (Additional file 1: Table S10). In the biosynthesis of unsaturated fatty acids, 22 DEGs were identified, including one stearoyl-ACP desaturase (SAD), 18 omega-6 desaturase (FAD2), and three omega-3 desaturase (one for FAD3, one for FAD7 and one for FAD8) genes. There were no related single genes encoding FAD4 and FAD5 detected in safflower seeds. Based on the expression analysis between the two species, *SAD* (Chr9G0227700) and *FAD2* (Chr8G0104100, Chr10G0038600) were highly expressed at 10DAF (FPKM > 100), and they may have important effects on the unsaturated fatty acid content during safflower seed development. In TAG synthesis, five DEGs were identified. Diacylglycerol acyltransferase (DGAT: Chr11G0235800) had a high expression in the rapid accumulation phase of oil, indicating its key role in TAG. In oil body formation, the increase in the expression level of oil body proteins can be attributed to the rapid accumulation of TAG. Five DEGs that encode oil body proteins were identified, among which oleosin (OLE: Chr1G0007600, Chr9G0052400, Chr11G0008500) had very high expression at two developmental stages, which is also consistent with the expression pattern of fatty acid and TAG synthesis genes. The accuracy and reproducibility of RNA-Seq results were confirmed by selecting six differential genes associated with safflower lipid synthesis for qRT-PCR analysis (Fig. 8). The relative expression of qRT-PCR was consistent with the data from RNA-Seq between the two products.

## Discussion

### Genetic diversity and population structure of 605 safflower germplasm

Phenotypic variation is an important component of genetic variation. The fatty acid composition of 605 safflower germplasm was evaluated. Fatty acid compositions, such as oleic, linoleic, and oil contents, varied considerably among safflower varieties, and the highest negative correlation was found between linoleic and oleic acids (*r* =  − 0.93***). This condition may be influenced by the environment and genes [21]. PCA results showed that oleic acid and linoleic acid were representative variations of germplasm. A total of 26 germplasm with linoleic acid content (> 78%) and 20 germplasm with oleic acid content (> 20%) were identified within the core germplasm, thus laying the foundation for the breeding and improvement of safflower crops.

With the rapid development of biotechnology, plant researchers have identified a large number of germplasm resources for different plants. Therefore, these germplasm resources need to be developed for effective management and utilization. A total of 50 pairs of InDel markers were used to assess the genetic diversity of 605 safflower germplasm. On average, the values of I and PIC were 0.553 and 0.311, respectively. the average I and PIC values for SSR markers were 0.49 and 0.32, while the average I and PIC values using SCoT markers were 0.43 and 0.61[22]. However, the PIC values in the present study were comparatively lower possibly because of the origin of the germplasm, its quantity, and the type of molecular markers used.

Structural analysis based on Bayesian models is widely used to infer hidden population structure in plant species [23]. In this study, we identified three subpopulations and one mixed population within 605 safflower germplasm, whose structural patterns have been validated by PCoA analysis (Fig. 3C). Based on AMOVA, genetic diversity within populations was more pronounced than between populations, and most of the genetic variation was present within populations. This finding is consistent with other results [24]. Moreover, according to the criteria proposed by Wright [25], *F*_*ST*_ < 0.05 indicates small genetic differentiation, 0.05 < *F*_*ST*_ < 0.15 indicates moderate genetic differentiation, 0.15 < *F*_*ST*_ < 0.25 indicates large differentiation, and *F*_*ST*_ > 0.25 indicates very large genetic differentiation. The obtained *F*_*ST*_ values indicate the presence of moderate or very large genetic differentiation, providing efficient genetic resource for the selection and breeding of new safflower cultivars.

In addition, considering the lack of genomic information, association studies for the quantitative traits of safflower oil have not been reported. In the present study, the GLM model was used for 11 oil traits in 605 safflower germplasm for association analysis. GLM analysis showed that 32 InDel loci were associated with safflower fatty acids. For example, the loci 33 locus, which is associated with 11-eicosenoic acid trait marker, explained 4.26% of the variation. These InDel loci may be useful for future genome-wide association analysis and will contribute to the future marker-assisted breeding of safflower.

### Combination of phenotypes and genotypes improves the reliability of constructing core germplasm repositories

Cultivated plants need to be conserved by establishing core germplasm for the optimal management and utilization of their genetic resources. Phenotypic data or molecular markers are often used to construct core germplasm. Phenotypic data are susceptible to external environmental factors and have a certain degree of inaccuracy, whereas molecular markers can effectively reduce external influences and provide more stable results. In order to obtain the maximum amount of alleles and genetic diversity in core germplasm, phenotypic data and genotypes should be combined to construct the core germplasm. As different strategies lead to different sets of cores, there are currently three common approaches: MSTRAT, Power Core and Core Hunter. The MSTRA and Power Core strategies select diverse incoming samples by optimising the allelic richness of the collection, while Core Hunter is not only able to select significantly smaller subsets of cores, but also these subsets retain all the unique alleles in the collection. Boccacci et al. compared the best subset of hazelnut core germplasm constructed based on ten SSR markers and using a range of different maximization strategy (MSTRAT, Power Core and Core Hunter) approaches, which found that the core hunter strategy was optimized at the same weights as the other strategies [26].

Among the core germplasm constructed for safflower, Kumar et al. reported that six independent core libraries (CC1-CC6) were created by POWERCORE and MSTRAT using molecular marker and phenotypic data from two seasons in safflower[7]. Here, 50 pairs of InDel markers were combined with 11 oil traits to construct the core germplasm library of 214 safflower germplasm using Core Hunter 3. The advantage of developing smaller core sets has been demonstrated previously in plants such as Indian mustard and Chinese fir [27–29]. The results revealed that all six genetic diversity parameters of the core germplasm were higher than the original germplasm at the molecular level. At the level of 11 quantitative traits, all of them met the evaluation criteria of MD% < 20% and CR > 80% [20]. Therefore, the core germplasm of safflower in this study can represent the original germplasm well, which makes an important contribution to improving the management of genetic resources of safflower germplasm and providing quality material for future research.

### Key genes associated with fatty acid biosynthesis and oil accumulation

The mechanistic process of oil biosynthesis mainly involves three steps, namely, fatty acid biosynthesis from scratch, TAG assembly, and oil body formation. In this study, key genes for FA biosynthesis and oil accumulation were identified during safflower seed development (Fig. 7). High expression of gene KAS II is required to enhance oleic acid precursor C18:0-ACP after the 10 DAF. SAD catalyzes the generation of C18:0-ACP to C18:1-ACP, which is a rate-limiting step in the formation of unsaturated fatty acids, while members of the FAD family have important roles in the biosynthesis of linoleic and linolenic acids [30, 31]. The expression of *SAD* gene (Chr9G0227700) was upregulated, whereas the expression of *FAD2* gene (Chr8G0104100) was downregulated in LL varieties in seed development at 10DAF, which may be essential for the biosynthesis of oleic acid from safflower. This regulatory mechanism is similar to the results of comparative transcriptome analyses of high- and low-oil Camellia oleifera[32]. In addition, the analysis based on RNA-seq data showed high expression of *FAD2* and low expression of *FAD3*, which is consistent with the small amount of C18:3 levels in safflower seed oil. However, *FAD3* was specifically expressed in perilla with C18:3 levels up to 50% or more. Therefore, FA biosynthesis is a conserved regulatory mechanism.

Most TAGs are stored in oil bodies consisting of a TAG core surrounded by a monolayer of phospholipids containing oil body membrane-associated proteins, such as oil body proteins, caleosin, and steroleosin) [33]. TAG assembly mainly involves GPAT, LPAAT, PAP, and DGAT, while DAG is involved in the TAG synthesis pathway, and it is the last enzyme that plays a key role in catalyzing TAG production in many oilseed crops [34]. In the present study, only one *DGAT* gene was identified, and the highest expression was reached at 10 DAF. In addition, OLE is the main protein of the oil body and participates in maintaining the size and stability of the oil body [35]. The expression level of OLE in Arabidopsis seeds was higher in seeds with high oil content than in seeds with low oil content[36]. In the present study, three *OLE* genes were identified with very high and increasing expression levels. Similarly, the trend of OLE expression in developmental seeds was consistent with other studies [37]. Therefore, the high oil accumulation in safflower seeds may be related to the high expression levels of *DGAT* and *OLE*.

## Conclusions

In the present study, 11 quantitative traits were used to evaluate the phenotypic diversity of 605 collected safflower germplasm, and 50 pairs of InDel markers were used to evaluate genetic diversity and population structure. A core germplasm containing 214 cultivars was subsequently constructed, and their evaluation indicators showed that the core germplasm was well representative of the original germplasm, thus providing an important genetic resource for the subsequent analysis. By using transcriptome sequencing, differential genes in the transcriptomes of high and low linoleic safflower cultivars at two stages of seed development were identified, and 47 candidate genes associated with unsaturated fatty acid biosynthesis and oil accumulation were obtained. The results of this study will be valuable for future research and breeding efforts.

## Materials and methods

### Plant materials and samples collection

All the 605 safflower germplasm resources were provided by the Oil Crops Research Institute of Chinese Academy of Agricultural Sciences, Wuhan, China. The plants were grown in Bole safflower base, Xinjiang. The planting density was 20 cm and spaced 40 cm apart, and the space was managed uniformly in the field. Mature open-pollinated seeds of the natural population were harvested and desiccated for the analysis of fatty acid contents (three repeats of each sample). At 10 and 20 DAF, three biological replications were collected, frozen in liquid nitrogen, and then stored at − 80 °C for transcriptome sequencing. We declare that the research programme complies with relevant institutional, national and international guidelines and legislation, and we have permission to collect safflower seeds.

### Fatty acid composition and oil content determination

Fatty acids were identified and quantified in fatty acids by measuring fatty acid methyl ester (FAME) by using gas chromatography-mass spectrometry (GC–MS, Agilent 890b-5977, China). A 40–50 mg sample was placed in a 15 mL glass tube, and 4.5 mL of sulfuric acid: methanol solution (volume ratio = 5:100) and 0.438 mg of heptadecanoic acid (C17:0, Cat No. H3500, Sigma-Aladtich, USA) dissolved in chloroform were added. The mixture was held in a water bath at 85 °C for 2 h. After cooling at room temperature, 3 mL of ultrapure water and 3 mL of n-hexane were added, and the mixture was shaken and mixed well. Afterward, centrifugation (1,000 rpm, 8 min) was carried out with a 0.45-μm organic-phase filter into a 2-mL chromatographic sample bottle [38].

For the GC–MS conditions, helium gas with a purity of 99.99% was selected at a carrier flow of 1 mL/min with a split flow of 20 mL/min. The initial temperature was maintained at 170 °C for 1 min, and then gradually increased to 230 °C at a rate of 3 °C/min and held for 3 min. Finally, the mixed standard solution of 37-component fatty acid methyl (Cat. no. DRE-A50000091HP, Laboratory of the Government Chemist, Britain) and sample solution were successively subjected to GC–MS to determine the chromatographic peak area of each fatty acid response.

The absolute fatty acid content was determined according to the national standard (GB 5009.168–2016) of the People’s Republic of China.

The calculation formula for the fatty acid absolute content is as follows:

$${X}_{i}={F}_{i}\times \left({A}_{i}/{A}_{C12}\right)\times \left({C}_{C17}\times {V}_{C17}/0.9507m\right)\times 100$$

*X*_*i*_ is the absolute content of fatty acid (g/100 g), *F*_*i*_ is the response factor of fatty acid methyl ester, Ai is the peak area of fatty acid methyl ester in the sample, *A*_*C17*_ is the peak area of heptadecanoic acid (C17) internal standard solution added to the sample, *C*_*C17*_ is the peak area of the heptadecanoic acid (C17) with a volume of 4.38 mg/mL, *V*_*C17*_ is the volume of heptadecanoic acid (C17) with a volume of 0.1 mL, *0.9507*^*–1*^ is the conversion coefficient of heptadecanoic acid to heptadecanoic acid methyl ester, *m* is the sample mass (mg), and 100 is the coefficient that converts the content to the content per 100 g of sample. The calculation formula of *F*_*i*_ is ${F}_{i}=\left({C}_{si}/{A}_{C17}\right)\times \left({A}_{si}/{C}_{C17}\right)$, where *C*_*si*_ is the concentration of fatty acid methyl ester in the 37-component fatty acid methyl mixture, *C*_*C17*_ is the concentration of heptadecanoic acid methyl ester in the 37-component fatty acid methyl mixture, *A*_*C17*_ is the peak area of heptadecanoic acid, and *A*_*si*_ is the peak area of fatty acid methyl ester. The calculation formula of oil content is ${X}_{Total Fat}=\sum {X}_{i}\times {F}_{FAMEi-TG}$, where *X*_*i*_ is the absolute content of fatty acid, and *F*_*FAMEi-TG*_ is the coefficient of conversion of fatty acid methyl ester to triglyceride.

### Genetic diversity, genetic structure, and correlation analysis of safflower germplasm

According to our previous results, 50 mapped InDel makers were selected (Additional file 1: Table S3). The observed number of alleles, effective number of alleles, Shannon’s, PIC, and heterozygosity were calculated using POPGENE version 1.3.2 software [39, 40].

The genetic structure of safflower was analyzed using Structure version 2.3.4 to determine the number of clusters (*K*) and calculate the Q value for analysis [41]. For the parameter settings, *K* was varied from 1 to 15, in which each *K* value was repeated for 10 times, the length of burn-in-period was 50,000, and the number of MCM Reps after Burnin was 500,000. The results were submitted to the website (http://taylor0.biology.ucla.edu/struct_harvest/) to determine a suitable *K* value. The run with the maximum likelihood was applied to subdivide the accessions into different subgroups with the membership probability threshold ≥ 0.70 as well as the maximum membership probability among the subgroups. Accessions with membership probabilities < 0.70 were retained in the admixed group (AD). The results of STRUCTURE were displayed with Distruct version 1.1 [42].

The relationship between quantitative trait and InDel genotype data was calculated using Tassel version 3.0 [43]. For the generalized linear model (GLM) method, population structure information (Q-matrix) was used as a covariate. The significance of associations between loci and traits was determined based on the P values (P < 0.01), which were calculated using statistical models, and the phenotypic variance explained by the significant loci was calculated.

### Core germplasm construction

The core germplasm of safflower was constructed using Core Hunter version 3.0 software [44]. The number of individuals selected for the sampling intensity was 20%. The analysis was carried out based on the average entry-to-nearest-entry distance, Gower’s distance for InDel marker data, and modified Roger’s distance, Cavalli-Sforza, and Edwards distance for quantitative trait data. Edwards distances were calculated. The representativeness of the core germplasm was tested in terms of the mean, extreme deviation, variance, and coefficient of variation [20].

### Total RNA extraction, cDNA library preparation, and transcriptome sequencing analysis

The RNA was isolated using a plant RNA extraction kit (Huayueyang, Beijing, China) following the instructions of the manufacturer. The concentration and purity of each RNA sample were determined using NanoDrop ND-2000 (NanoDrop, Wilmington, DE, USA), and its integrity was assessed using Agilent 2100 with RIN number > 7.0. For RNA sequencing, 12 cDNA libraries were constructed, and paired-end sequencing was performed on the Illumina NovaSeq 6000 platform (Annoroad, Beijing, China) according to the vendor’s recommended protocol.

Cutadapt [45] was used to remove reads that contain adaptor contamination, low quality reads, and undetermined base. Clean data analysis was performed using Bowtie version 2.3.4.3. Sequences were aligned with the safflower genome (https://safflower.scuec.edu.cn). The aligned sequences were quantified for gene expression using RSEM version 1.3.1, and the amount of gene expression was expressed in FPKM [46].

### Differentially expressed genes (DEGs), gene function annotation, and enrichment analysis

DEGs were detected using the R package of DESeq2 version 1.30.1 with fold-change > 1 or <  − 1 and an adjusted p value < 0.05 [47]. Kyoto Encyclopedia of Genes and Genomes (KEGG) pathway enrichment analyses for the DEGs were carried out using GOstats version 2.60.0 and GSEABase version 1.56.0 [48].

### Gene expression analysis using quantitative real-time PCR (qRT-PCR)

Gene-specific and internal control primers for genes related to lipid biosynthesis are shown in Table S10, in which the β-actin gene of walnut was used as a reference gene.

Approximately 1.0 μg of RNA was reverse-transcribed into first-strand cDNA by using MonAmp 2 × Taq Mix for qPCR (Mona, China). qPCR was carried out in the CFX Connect Real-Time PCR Detection System (Bio-Rad, USA) by using 2 × Universal Blue SYBR Green qPCR Master Mix (Servicebio, Wuhan, China). The experimental conditions were set as follows: 40 cycles at 95 °C for 30 s (predegeneration), 95 °C for 15 s (denaturation), 58 °C for 10 s (annealing), and 72 °C for 30 s (extension). The mRNA expression level of the genes was calculated with the 2 − ΔΔCt method. Three technical replicates were performed (Additional file 1: Table S11).

### Statistical analysis

F-statistics, including F_st_, hierarchical AMOVA, and the pairwise *F*_*st*_, were carried out using GenAlEx version 6.502. The mean, maximum, and minimum value and the coefficient of variation of fatty acid composition and oil content were analyzed using SPSS 19. The formula for calculating the coefficient of variation is CV(%) = (SD /MN) × 100, where SD is the standard deviation, and MN is the mean. The diversity index is represented by the Shannon–wiener index, $H{\prime}=\sum {P}_{i}Ln{P}_{i}$, where* P*_*i*_ is the probability of an *i*-th value. The quantitative traits were divided into 10 levels, 1 level < *X-2σ*, 10 level ≥ *X* + *2σ*, phase difference of each intermediate stage 0.5σ, where *σ* is the standard deviation.

PAST software version 3.14 was used to construct a dendrogram through the unweighted pair group arithmetic average (UPGMA) method and by applying the Euclidean[49]. To measure the stability of the computed branches, a statistical bootstrap analysis was conducted with 1000 resampling replicates. All trees are visualized with the iTOL webtool version 5 [50].

### Supplementary Information

**Additional file 1: Table S1-S10.** 

**Additional file 2:** **Fig. S1. **Differences in the fatty acid content of safflower seeds at different developmental stages. DAF, days after flowering.

## Data Availability

The raw sequence data from this study have been deposited in the publicly accessible National Genomics Data Center (NGDC, https://ngdc.cncb.ac.cn/) database as accession number PRJCA017071. The datasets supporting the conclusions of this article are included within the article and its additional files. The datasets used and/or analyzed during the current study are available from the authors on reasonable request (Rui Qin, qinrui@scuec.edu.cn).

## Abbreviations

α-PDHC: Pyruvate dehydrogenase alpha subunit
β-PDHC: Pyruvate dehydrogenase beta subunit
DHLAT: Dihydrolipoamide acetyltransferase
LPD: Dihydrolipoamide dehydrogenase
α-CT: Carboxyl transferase subunit alpha
β-CT: Carboxyl transferase subunit beta
BC: Biotin carboxylase
BCCP: Biotin carboxyl carrier protein
MCAAT: Malonyl-CoA ACP transacylase
ACP: Acyl carrier protein
KAS I, II, III: Ketoacyl-ACP synthase I, II, III
KAR: Ketoacyl-ACP reductase
HAD: Hydroxyacyl-ACP dehydrase
EAR: Enoyl-ACP reductase
SAD: Stearoyl-ACP desaturase
FAD2: Oleate desaturase
FAD6: Oleate desaturase
FAD3: Linoleate desaturase
FAD7: Linoleate desaturase
FAD8: Linoleate desaturase
FATB: Acyl-ACP thioesterase B
GPAT: Glycerol-3-phosphate acyltransferase
LPAAT: 1-Acyl-sn-glycerol-3-phosphate acyltransferase
PAP: Phosphatide phosphatase
DGAT: Diacylglycerol O-acyltransferase
PDAT: Phospholipid: diacylglycerol acyltransferase
OB: Oil body
OLE: Oleosin
CLO: Caleosin
