# Supplementary material for: Identification of genes associated with fatty acid biosynthesis based on 214 safflower core germplasm
Source: BMC Genomics. 2023 Dec 11;24:763. doi: 10.1186/s12864-023-09874-5 (PMC10712096; doi:10.1186/s12864-023-09874-5)
Supplement: Supplementary file 2 — Additional file 2: Fig. S1. Differences in the fatty acid content of safflower seeds at different developmental stages. DAF, days after flowering. [file 12864_2023_9874_MOESM2_ESM.docx]

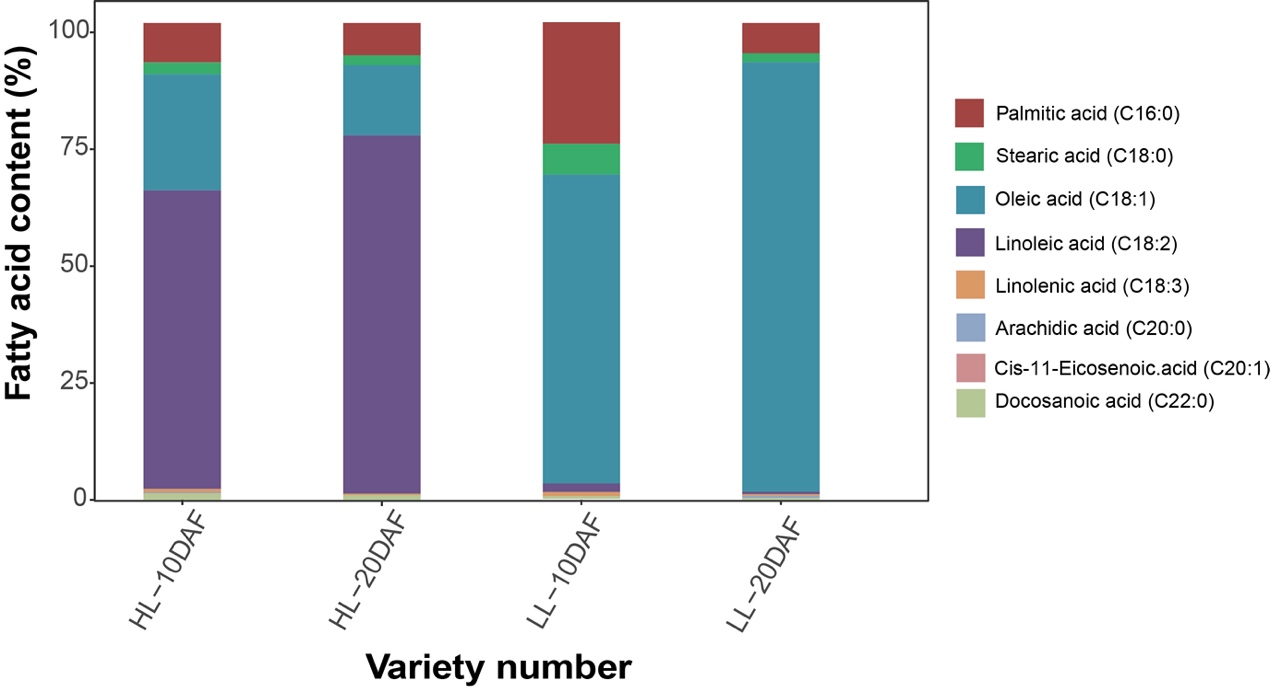


**Fig.S1.** Differences in the fatty acid content of safflower seeds at different developmental stages. DAF, days after flowering.
